# A mechanism for deviance detection and contextual routing in the thalamus: a review and theoretical proposal

**DOI:** 10.3389/fnins.2024.1359180

**Published:** 2024-02-29

**Authors:** Carmen Varela, Joao V. S. Moreira, Basak Kocaoglu, Salvador Dura-Bernal, Subutai Ahmad

**Affiliations:** ^1^Psychology Department, Florida Atlantic University, Boca Raton, FL, United States; ^2^Department of Physiology and Pharmacology, State University of New York (SUNY) Downstate Health Sciences University, Brooklyn, NY, United States; ^3^Center for Connected Autonomy and Artificial Intelligence, Florida Atlantic University, Boca Raton, FL, United States; ^4^Center for Biomedical Imaging and Neuromodulation, Nathan S. Kline Institute for Psychiatric Research, Orangeburg, NY, United States; ^5^Numenta, Redwood City, CA, United States

**Keywords:** deviance detection, corticothalamic, burst, thalamus firing mode, predictive processing, layer 6, cortical feedback

## Abstract

Predictive processing theories conceptualize neocortical feedback as conveying expectations and contextual attention signals derived from internal cortical models, playing an essential role in the perception and interpretation of sensory information. However, few predictive processing frameworks outline concrete mechanistic roles for the corticothalamic (CT) feedback from layer 6 (L6), despite the fact that the number of CT axons is an order of magnitude greater than that of feedforward thalamocortical (TC) axons. Here we review the functional architecture of CT circuits and propose a mechanism through which L6 could regulate thalamic firing modes (burst, tonic) to detect unexpected inputs. Using simulations in a model of a TC cell, we show how the CT feedback could support prediction-based input discrimination in TC cells by promoting burst firing. This type of CT control can enable the thalamic circuit to implement spatial and context selective attention mechanisms. The proposed mechanism generates specific experimentally testable hypotheses. We suggest that the L6 CT feedback allows the thalamus to detect deviance from predictions of internal cortical models, thereby supporting contextual attention and routing operations, a far more powerful role than traditionally assumed.

## 1 Introduction

The thalamus constitutes a central forebrain hub where inputs from brainstem neuromodulators and neocortical layer 6 (L6) influence the routing and processing of information by regulating the depolarization level of thalamic cells (Destexhe et al., 1996; Briggs and Usrey, 2008; Saalmann, 2014; Dolleman-van der Weel et al., 2019; Jaramillo et al., 2019; Usrey and Sherman, 2019; Antunes and Malmierca, 2021). The membrane potential of thalamic cells determines two fundamentally different modes of response to inputs: tonic and burst firing (Jahnsen and Llinás, 1984; Sherman, 2001a; Llinás and Steriade, 2006). The tonic firing mode predominates when thalamocortical (TC) cells are depolarized, when the firing rate varies directly with changes in the input, in alignment with the idea of a TC gate controlling information transmission in a linear manner. In contrast, burst firing occurs when cells are hyperpolarized, such as through inhibition mediated by GABAergic cells in the thalamic reticular nucleus (TRN), and it is a non-linear response mode that provides better input detectability than tonic firing (Crick, 1984; McCormick and Feeser, 1990; Guido et al., 1995; Sherman, 1996; Reinagel et al., 1999; Sherman, 2001a). While burst firing is more common during sleep, it has been reported during wakefulness in relation to attention and novelty, but how it contributes to those functions is unclear (Lesica and Stanley, 2005; McAlonan et al., 2008; Ortuño et al., 2014; Whitmire et al., 2016).

Several theoretical proposals (Hawkins et al., 2018; Lewis et al., 2019; Bennett, 2020; Antunes and Malmierca, 2021) and recent experimental results (Voigts et al., 2020; Clayton et al., 2021), suggest that L6 CT cells may represent predictions from internal models stored in the neocortex. Experimental results also suggest that one of the functions of L6 CT cells is to regulate the occurrence of burst in the thalamus (Ortuño et al., 2014; Kirchgessner et al., 2020). However, it is not known how the L6 regulation of thalamic bursts contributes to predictive processing. The “searchlight hypothesis” (Crick, 1984) laid out a potential mechanism for internal attention where an increase in TRN activity would inhibit TC cells to make them burst, transiently tagging attended information. A few years later, Mumford suggested that the thalamus contributes to noise removal and to the identification of relevant input features, functioning as an “active blackboard” that interprets incoming signals within the context of the outcomes of cortical computations transmitted via CT projections (Harth et al., 1987; Mumford, 1991; Worden et al., 2021). Nonetheless, no specific account was given as to how the organization of thalamic circuits and cellular firing mode could achieve these functions. These theories are consistent with the suggestion that L6 feedback projections represent cortical expectations sent to lower levels of the cortical processing hierarchy (Rao and Ballard, 1999; Friston, 2005, p. 200; Bastos et al., 2012; Keller and Mrsic-Flogel, 2018). However, there have been few attempts to refine these ideas in light of new evidence on the organization and function of thalamic networks (Pinault, 2004; Asilador and Llano, 2020; Antunes and Malmierca, 2021). As a result, the precise cellular mechanisms by which L6 CT projections implement top-down contextual and inferential computations to process inputs to the thalamus remain unclear, limiting the advance of predictive processing theories (Kogo and Trengove, 2015).

Here we review the functional organization of CT projections and introduce the hypothesis that L6 CT direct and indirect (via TRN) projections to TC cells implement a predictive code for expected and unexpected input features (location, context, stimulus properties). We will use the term context broadly to denote top-down influences (represented by cells within a cortical module, such as a column or area) encompassing prior knowledge (stored in internal mental models) that animals utilize to make predictions about the environment. The mechanism we propose allows for the utilization of TC firing mode to evaluate incoming input in relation to cortical predictions. This enables top-down influences that could facilitate location-, feature-, and other context-based inference and attention (Hawkins et al., 2018; Lewis et al., 2019; Antunes and Malmierca, 2021). The circuit architecture and biophysical mechanisms required for this control are present across all thalamic nuclei, from sensory to cognitive regions. Thus, the mechanism proposed here affords powerful control over inputs to neocortical and non-neocortical regions connected with the thalamus (Jones, 1998; Martel and Galvan, 2022).

In the first part of this manuscript, we discuss the literature that supports this hypothesis. In the second part, we use a computational model to demonstrate a specific instantiation of the proposed hypothesis, where the control of firing mode could facilitate dendritic-specific triggering of bursts in individual TC cells. Such a mechanism could enable context-dependent input detection at the single cell level.

## 2 Functional organization of thalamocortical circuits

### 2.1 Corticothalamic circuit motifs inhibit functionally misaligned thalamocortical cells

**Figure 1:**
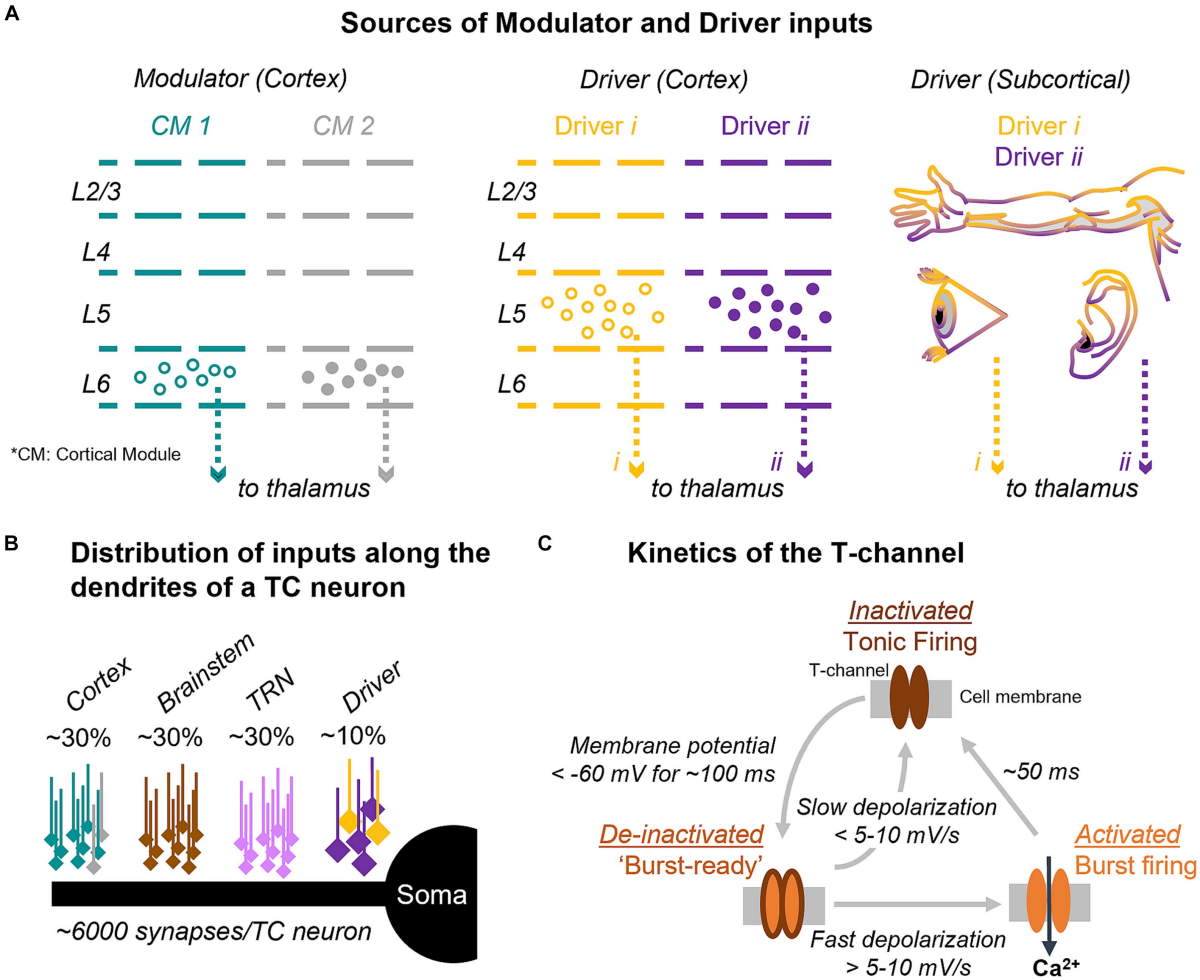
Input and synaptic architecture controlling T-channel dynamics and firing mode in thalamocortical cells. **(A)** Thalamic input from cortical modulators (left) and from cortical and subcortical drivers (center and right) are topographically and functionally organized. **(B)** Diagram of the distribution of synaptic inputs along the dendrites of thalamocortical (TC) cells. Percent values indicate the estimated fraction of synapses from each input. **(C)** T-channels are inactivated at resting membrane potentials (top), which promotes tonic firing. Hyperpolarization of the cell below −60 mV de-inactivates the channels and primes them for activation (“burst-ready,” left); if a strong input arrives, the T-channels activate transiently leading to a low-threshold calcium spike with associated sodium-potassium action potentials (right). CT, corticothalamic; TC, thalamocortical; TRN, thalamic reticular nucleus; CM, cortical module (e.g., column, area).

The basic premise that we introduce here is that the circuit architecture of thalamic inputs and the firing mode of thalamic cells may support a mechanism for detecting deviance from cortical predictions. Inputs to the thalamus are classified into drivers, which determine the receptive field properties of TC cells, and modulators, which influence the excitability and functional state of the cells, such as their firing mode (Sherman and Guillery, 1998; Figure 1A). Drivers originate in subcortical afferents from sensory organs or in layer 5 of the cortex. Modulators, on the other hand, originate in several areas, including cortical L6, and contribute most of the synapses on TC cells (Figure 1B). Modulators produce relatively small postsynaptic potentials that are unlikely to trigger spikes but can last hundreds of milliseconds, influencing voltage-and time-dependent channels such as the T-type (“transient”) calcium channels that mediate burst firing (Figure 1C). One of the general functions of modulators is to switch thalamic cells between tonic and burst firing modes (McCormick and von Krosigk, 1992; Bista et al., 2012). Unlike modulator inputs from the brainstem, which present a loose topography and are thought to switch the firing mode of TC cells as a function of general arousal states, like wakefulness and sleep (Briggs and Usrey, 2011; Varela, 2014), the L6 CT inputs are topographically and functionally constrained and could regulate the TC firing mode under context-or stimulus-specific conditions.

#### 2.1.1 Cortical regulation of thalamic excitability is determined by circuit topography

Top-down L6 CT excitatory projections target distal regions of TC cell dendrites, while also branching to provide disynaptic GABAergic input to TC cells via TRN and local interneurons (Deschênes et al., 1998; Kimura et al., 2007; Landisman and Connors, 2007; Mease et al., 2014; Crandall et al., 2015). The L6 excitatory (direct) and inhibitory (indirect) projections are arranged both in closed-and open-loop systems (Land et al., 1995; Deschênes et al., 1998; Lam and Sherman, 2010; Brown et al., 2020; Rockland, 2021; Shepherd and Yamawaki, 2021). Although the anatomical organization of the two branches of the CT projection at the single cell level is still unclear, the L6 excitatory and inhibitory footprints to individual TC cells can originate from areas located tens of micrometers apart or more in L6 (Lam and Sherman, 2010). This suggests that CT input from functionally different cortical cells may converge on TC cells through the direct and indirect pathways (Buxhoeveden and Casanova, 2002). This circuit arrangement is interesting because if L6 represents cortical predictions as suggested by theory (Hawkins et al., 2018; Lewis et al., 2019; Bennett, 2020; Antunes and Malmierca, 2021) and experimental results (Voigts et al., 2020; Clayton et al., 2021), predictions from different CT cells would converge on a given TC cell and regulate its membrane potential (and firing mode) through a push-pull mechanism (Hirsch et al., 2015).

The idea that the direct and indirect (through TRN) L6 CT inputs to a given TC cell are topographically different is supported by *in vivo* evidence (reviewed below) from several sensory systems. For example, the sign of the correlation between spikes from cell pairs recorded in L6 of the primary visual cortex and in the dorsal lateral geniculate nucleus (dLGN) is determined by retinotopy. When the receptive fields of the L6-dLGN pair are close in visual space (within a few degrees of the visual field), L6 and dLGN spikes are either positively correlated (suggesting L6 excitation of dLGN) or negatively correlated (suggesting L6 inhibition of dLGN via TRN). However, with larger separations between the receptive fields, negative spike correlations are more common (Tsumoto et al., 1978; Wang et al., 2006, 2018; Jones et al., 2012). Optogenetic stimulation of L6 CT feedback has confirmed this pattern of retinotopic organization. Consistent with the older studies, optogenetic stimulation of L6 CT projections resulted in dLGN excitation or inhibition when cortical and dLGN receptive fields were within 30 degrees of visual field, whereas inhibitory effects were more common at greater distances (Born et al., 2021).

Similar observations have been documented in the somatosensory and auditory systems. Activation of L6 increased sensory responses in somatotopically equivalent regions of thalamus, but it decreased sensory responses in non-homologous thalamic barreloids (Temereanca and Simons, 2004; Li and Ebner, 2016). In the auditory thalamus, cortical inactivation decreased the auditory responses of tonotopically aligned thalamic neurons and it increased the responses of thalamic neurons with different frequency tuning (Zhang et al., 1997), consistent with anatomical data on the tonotopic organization of corticothalamic projections (Kimura et al., 2007).

**Figure 2:**
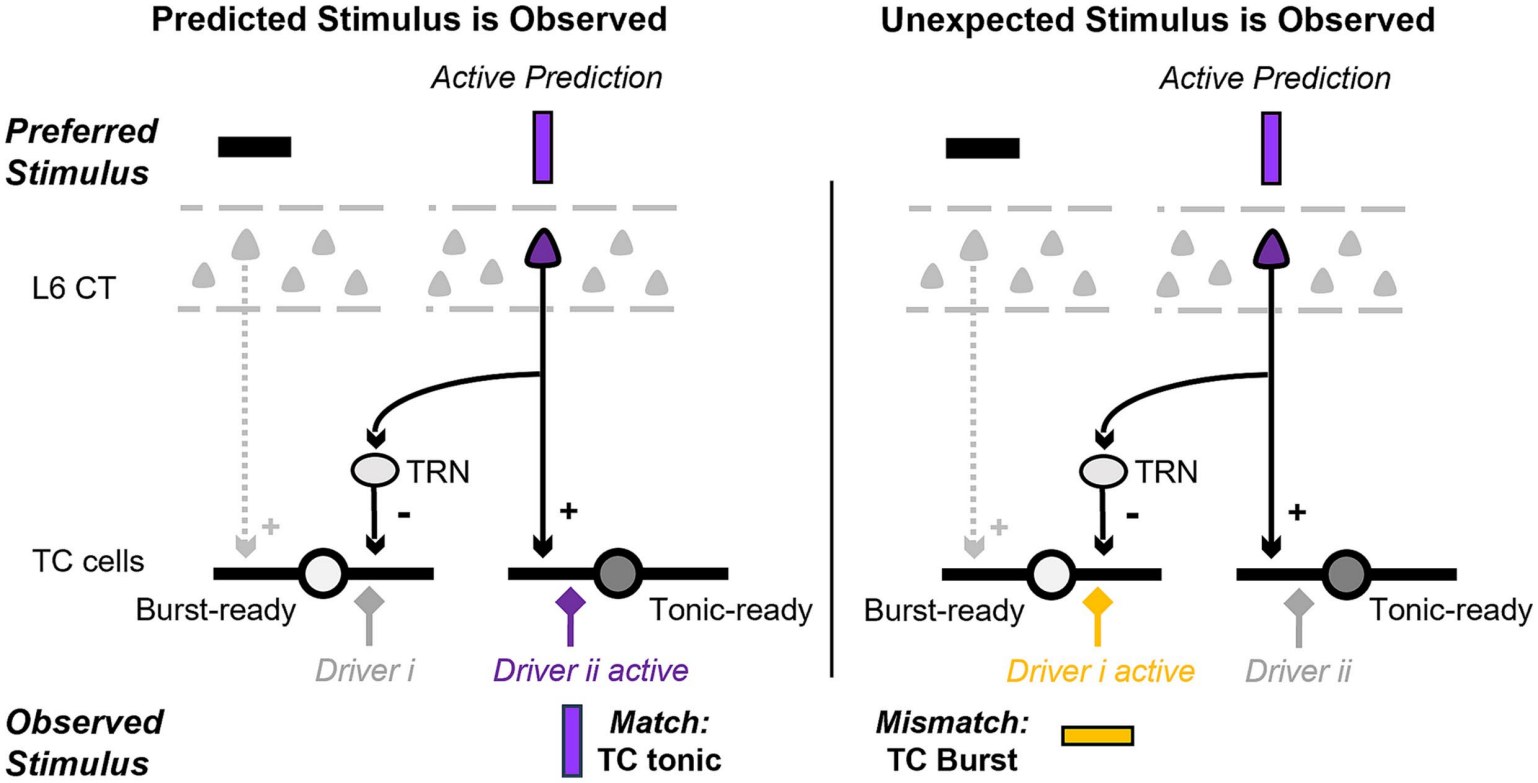
Pictorial representation of the proposed hypothesis and its predictions at the single cell level. We review evidence suggesting that the layer 6 (L6) corticothalamic (CT) neurons that provide direct excitatory and indirect inhibitory input to thalamocortical (TC) cells have different functional properties. At the single cell level, this circuit could depolarize and hyperpolarize specific dendrites and facilitate the discrimination of observed inputs that are a match (left; driver ii) versus a mismatch (right; driver i) to the cortical prediction.

These results suggest that L6 CT excitation (and inhibition in the visual system) occurs at closely aligned topographic locations, and that L6 inputs topographically misaligned with their TC targets primarily evoke inhibition. In other words, activation of L6 CT cells that contribute to stimuli represented at a specific topographic location depolarizes cells in the corresponding location of the thalamus, and the fraction of hyperpolarized TC cells increases with topographic distance. From a predictive processing perspective, this could provide a circuit mechanism where, when a specific L6 cell ensemble is active, it simultaneously depolarizes thalamic locations where input is expected and hyperpolarizes the locations where input is unexpected, in line with cortical inferences (Figure 2).

#### 2.1.2 Functional tuning similarity dictates CT regulation of TC excitability

A related question is whether the L6 CT effect (excitation, inhibition) on TC cells is contingent upon similar response properties between L6 and TC cells, such as similar tuning curves to the features of sensory stimuli. For example, if an L6 and a TC cell overlap in topographic location, does their preferred tuning determine whether L6 exerts an excitatory or inhibitory effect on the TC cell? In the auditory and somatosensory systems, the evidence is consistent with the topographic organization: excitation when the tuning properties are similar and inhibition otherwise. A series of experiments in the bat’s auditory system (reviewed in Suga and Ma, 2003), demonstrated that when tuning properties differ between L6 and TC cells, the thalamic neuron’s response is inhibited at its preferred frequency but facilitated at other frequencies following electrical stimulation of L6. Conversely, cortical stimulation increased the response to the preferred sound frequency of tonotopically aligned TC cells, while reducing their response to non-preferred frequencies (Zhang and Suga, 2000). Likewise, in the somatosensory system, cortical stimulation increased TC responses to the preferred direction of whisker movement when the cells recorded in the cortex and thalamus exhibited similar angular tuning. However, the effect was inhibitory if the cells preferred different angular deflections of the whisker (Li and Ebner, 2007; Li and Ebner, 2016).

The evidence from the visual system is generally consistent with the findings in the auditory and somatosensory systems. Recordings of pairs of TRN and dLGN cells suggest increased inhibition of dLGN when the spatial and frequency tuning curves of the pair are different (Osaki et al., 2018). Furthermore, manipulation of L6 activity resulted in changes in firing rates (increase or decrease) in TC cells with receptive fields that were either parallel or perpendicular to the orientation of the manipulated L6 cells’ receptive field (Wang et al., 2006, 2018). This finding concerning orientation tuning is consistent with the earlier discussed results on retinotopy, which showed strong (positive and negative) spike correlations in topographically aligned L6-dLGN pairs (Tsumoto et al., 1978; Wang et al., 2006, 2018; Jones et al., 2012). On the other hand, a pharmacologically-induced increase in L6 activity produced opposite effects in the thalamus with respect to ON–OFF receptive field properties: if the L6 and dLGN cells had overlapping receptive fields of the same sign (both ON or both OFF), L6 activity was associated with an increase in the proportion of bursts in dLGN (suggestive of dLGN inhibition), but if the fields had opposite signs (ON and OFF) L6 led to a decrease in the number of bursts (suggestive of an excitatory effect) (Wang et al., 2006). It is possible that anesthesia effects on TRN or local interneuron networks may have reversed the sign of L6 effects on dLGN in this study compared to the other reports.

Another noteworthy line of evidence comes from the study of anticipatory firing in the somatosensory thalamus during active tactile discrimination (Pais-Vieira et al., 2013). This study noted both increases and decreases in TC cell firing during the anticipatory period of the tactile task. In contrast, cells in the deep layers of the somatosensory cortex primarily exhibited an increase in firing rate, suggesting that anticipatory firing in deep cortical layers translates into both activation and inhibition within the TC cell population. Although the relation between anticipatory excitation or inhibition of TC cells and the tuning properties of CT cells was not examined, another study found that activation of motor cortex resulted in the activation of topographically aligned TC somatosensory cells (Lee et al., 2008). This suggests that the decrease in firing may occur in TC cells that are topographically or functionally misaligned with cortex.

Overall, the experimental evidence suggests that when L6 CT cells are active, they simultaneously facilitate the response of TC cells that have similar topography and tuning properties to the CT cells, and they also inhibit (potentially through their axonal branches to TRN) TC cells that represent a mismatch to the L6 tuning. One hypothesis to explain these observations is that the L6 CT direct and indirect projections implement a predictive code of expected and unexpected (deviant) features sent to TC cells to be evaluated against incoming input. A basic prediction of this hypothesis is that under L6 activation, the population of TC cells would consist of cells in tonic mode and cells in a “burst-ready” mode. At the single cell level, this could involve setting individual dendrites in tonic and burst-ready mode (Figure 2), a scenario we explore in section 3. Due to the de-inactivation of the T-current with hyperpolarization, the L6-mediated inhibition could lead to bursts in burst-ready TC cells (or dendrites) when the incoming input features are unexpected (i.e., novel compared to what is predicted by cortical models). As reviewed in the next section, burst firing in awake animals is indeed associated with attention and novelty.

### 2.2 Thalamocortical burst firing during awake behavior is associated with novelty

The evidence reviewed above suggests that L6 CT neurons inhibit TC cells with dissimilar topography and functional properties and could switch them to burst mode. T-type calcium channels responsible for burst firing are distributed along the dendrites and soma of TC cells (Parajuli et al., 2010; Chen et al., 2017) and can be in one of three states (Figure 1C): inactivated, de-inactivated, and activated. If the membrane potential is hyperpolarized below about −60 mV, the channels enter the de-inactivated state and are primed to become active, thus, “burst-ready.” Subsequent significant membrane depolarization switches T-channels to a transient (40–50 ms) active state, which generates an inward Ca^2+^ current. This current in turn can initiate a burst of somatic Na^+^-K^+^ action potentials with brief inter-spike intervals (~5 ms) between individual action potentials (Lu et al., 1995). Compared to the tonic mode, the burst mode is non-linear and provides increased signal-to-noise properties (Guido et al., 1995; Reinagel et al., 1999). This makes burst firing an efficient mode for indicating non-linear, discrete events, such as sudden changes in the environment or the detection of attended stimuli in the searchlight hypothesis. Furthermore, bursts have higher transmission efficacy at retinogeniculate and TC synapses (Swadlow and Gusev, 2001; Alitto et al., 2019), suggesting that they activate cortex more reliably than tonic spikes (Hu and Agmon, 2016). These properties have led to the proposal that thalamic bursts serve as a discrete attentional signal, or a “wake-up” call to the cortex (Crick, 1984; Reinagel et al., 1999; Sherman, 2001a,b). While this explanation cannot fully account for the function of bursts, given their higher occurrence rates during non-rapid-eye-movement (NREM) sleep (Hirsch et al., 1983; Weyand et al., 2001), it offers a hypothesis for their function during awake behavior.

Bursts have been reported in awake animals during sensory stimulation, suggesting a role in information processing. In general, the proportion of spikes in bursts in TC cells of awake animals is very low, averaging 1–2% (Guido and Weyand, 1995; Weyand et al., 2001; Massaux et al., 2004; Wang et al., 2007; Alitto et al., 2011), but the fraction can be close to 20% in higher order TC cells, those connected with higher order cortical areas (Ramcharan et al., 2005; Varela and Wilson, 2020). However, only these two studies compared burst rates directly between first order (FO, sensory) and higher order (HO) thalamic cells using extracellular electrophysiology, which necessarily relies on the pattern of inter-spike intervals to detect bursts and therefore cannot identify burst spikes directly (Lu et al., 1995; Massaux et al., 2004). In addition, average proportions can be misleading because bursts may occur transiently at high rates during specific behaviors, or in the presence of certain sensory stimuli. For instance, bursts represented 20–30% of the spikes in the somatosensory and auditory thalamus in response to whisker or auditory stimulation (Massaux et al., 2004), although their proportion was low with visual stimulation (Weyand et al., 2001). A key open question is to understand how the variance in the burst rates reported in the literature relates to bottom-up mechanisms, such as input properties, or to top-down processes such as contextual or attentional regulation. In particular, the modulation of TRN activity during covert attention (McAlonan et al., 2006, 2008) could be regulated by L6 activation.

The possibility that bottom-up mechanisms regulate bursts is supported by the finding of bursts after stimuli that are thought to hyperpolarize thalamic cells, such as stimuli with non-preferred features or those that engage the inhibitory surround (Weyand et al., 2001; Lesica and Stanley, 2004; Alitto et al., 2005; Wang et al., 2007). Bursts are also found following periods of sensory quiescence, in the early part of the response to sensory stimulation (Guido et al., 1992; Guido and Weyand, 1995; Alitto et al., 2005; Ortuño et al., 2014; Whitmire et al., 2016). The evidence is more limited regarding the role of CT projections in inducing context-or attention-dependent TC hyperpolarization and bursts. Yet, bursts are observed during behaviors in which the CT system may play a role, such as in dLGN cells after eye saccades (Guido and Weyand, 1995; Martinez-Conde et al., 2002), and in the somatosensory thalamus during whisker twitching, which is thought to be initiated by cortex (Fanselow et al., 2001).

We hypothesized in section 2.1 that the L6 CT projections to TRN can induce inhibition in functionally misaligned TC cells, which predicts that bursts would be more likely in TC cells that represent a mismatch to features (location, stimulus properties, context) represented by active L6 cells. That is, TC cells in “burst-ready” mode may provide a thalamic representation of what is unexpected based on inference from internal cortical models. This mechanism could explain the increase in bursts observed when sensory stimuli are novel compared to after a few presentations under similar attention conditions (Guido and Weyand, 1995; Ortuño et al., 2014). The convergence of L6 CT excitatory and functionally different indirect inhibitory inputs on single TC cells raises the intriguing possibility that a burst-ready predictive code could be implemented at the dendritic level to discriminate inputs (Figure 2), a possibility we assess computationally in Section 3.

## 3 Computational modeling illustrates how TC cells could use bursts to discriminate inputs

Setting specific dendrites in burst mode may provide a robust mechanism to discriminate inputs at the single cell level based on firing mode. Evidence from the mouse visual system shows that individual TC cells receive functionally distinct subcortical inputs on different dendrites (Morgan et al., 2016; Liang et al., 2018) and drivers of cortical and subcortical origin converge on higher order TC cells (Groh et al., 2014; Bickford, 2015). With synaptic inputs segregated on different dendrites, our hypothesis predicts that driver input on a “burst-ready” (hyperpolarized) dendrite will trigger a burst, while driver input to a non-hyperpolarized dendrite in the same TC cell will not trigger a burst or will instead produce tonic spikes, resulting in distinct TC outputs. We tested these predictions in a three-compartment (soma and two dendrites) model of a TC cell defined by Hodgkin-Huxley channel dynamics (see Section 5).

**Figure 3:**
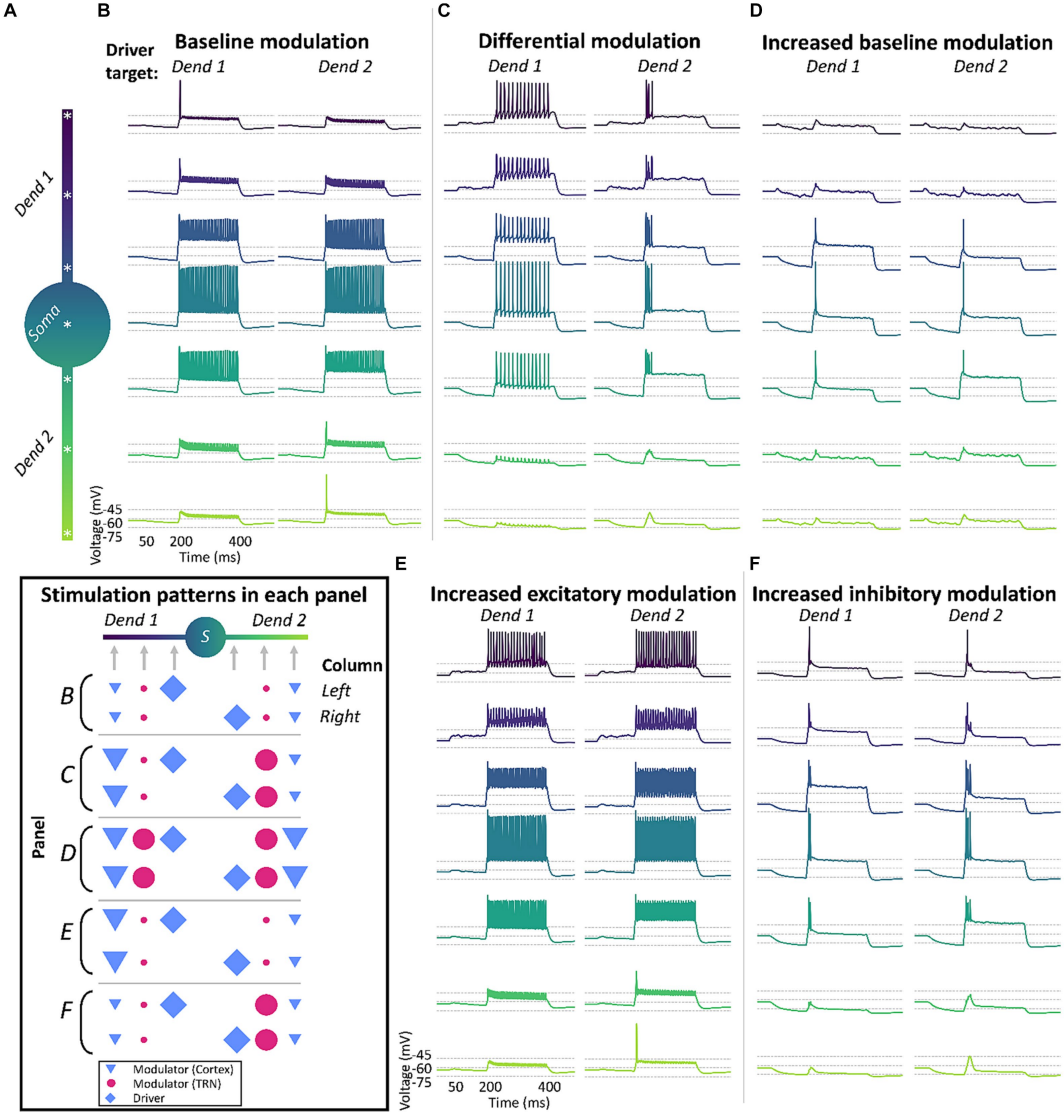
Differential modulation across dendrites enables dendrite-specific, tonic and burst firing in response to different driver inputs in a biophysical neuron simulation. TC model cells will respond differently based on the combination of driver (⬥) input and the strength of cortical (▼) and TRN (●) modulation across the dendritic tree. **(A)** Morphology and recording locations (*) in the 3-compartment TC cell model. **(B)** The model cell responds in tonic-mode under uniform “baseline” modulation from cortex and TRN, regardless of which dendrite receives the driver input (Dend 1 or Dend 2). **(C)** The TC cell fires in tonic or burst mode under differential modulation, with cortex and TRN providing stronger input to opposite dendritic branches. The difference in modulatory excitation and inhibition in each branch allows the dendritic tree of the cell to be in tonic-ready and burst-ready modes at the same time in different dendrites, responding differently depending on the source of driver inputs. **(D)** Model loses the ability to fire in different modes when there is a uniform increase in the strength of cortex and TRN modulation in both dendritic branches. **(E,F)** Absence of distinct firing modes when the cell is targeted with increased excitation **(E)** or inhibition **(F)** separately, regardless of driver input location. Inset: summary of input location and strength across the different conditions.

We investigated the effect of an excitatory, driver-like, input arriving at each of the two dendrites in a series of conditions and recorded the changes in membrane voltage across different regions in the TC cell model (Figure 3A). Each trace in Figures 3B–F represents the membrane voltage at each recording site. To match experimental data, the CT synapses were placed in the distal part of the model’s dendrites, the TRN synapses in middle regions, and the driver synapses were located in proximal areas of the dendrites (Wilson et al., 1984; Liu et al., 1995). In this condition, the cortical and TRN modulator inputs consisted of a cascade of artificial EPSPs (CT) or IPSPs (TRN) that lasted for 400 ms, with an average interval of 0.3 ms between each stimuli targeting the medial (half point of the dendrite length, or 0.5) and proximal (0.3) sectors of the dendrites. Synaptic models for AMPA, NMDA, GABA_A_, and GABA_B_ consisted of a double exponential function defined by rise and decay time constants. Modulator synapses on TC cells consisted of a combination of AMPA and NMDA conductances (70:30 ratio) to simulate CT inputs, and GABA_A_ and GABA_B_ conductances (50:50 ratio) to simulate synapses from TRN. Driver inputs consisted of a cascade of AMPA EPSPs, that started 150 ms after the modulatory inputs and lasted for 250 ms, and with individual PSPs delivered at 0.01 ms intervals. The driver inputs targeted the basal sectors of the dendrites (0.1). The geometry and ionic currents used to reproduce the biophysics of the TC cells are described in Table 1.

**Table 1:** Model geometry and conductance values for each compartment.

Geometry		Soma	Dendrites	Units
Length		17	600	μm
Diameter		17	2	μm
Axial resistance		150	150	Ohms*cm
Capacitance		0.878	1.68576	μF/cm^2^
**Conductances**		**Soma**	**Dendrites**	**Units**
IH	gH	20	0	mS/cm^2^
IK	gK	10	10	mS/cm^2^
INa	gNa	9	9	mS/cm^2^
IA	gA	–	1	mS/cm^2^
IT	gT	660	3.9	mS/cm^2^
	Shift	2	2	mV
IKleak	gKL	–	75	μS/cm^2^
	e	–	−105	mV
ILeak	gL	87.5	175	μS/cm^2^
	e	−50	−50	mV

First, we characterized the response of the cell to driver inputs when both dendrites were under the same modulatory influence, consisting of equal levels of sustained low-amplitude cortical excitation and TRN inhibition that targeted both dendrites uniformly (Figure 3B). We found that in this condition there was a mild hyperpolarization of the TC cell, which was not sufficient to de-inactivate a substantial number of T-channels. Therefore, when an excitatory driver input arrived at either dendrite, it produced virtually identical tonic responses in the TC cell, regardless of the dendrite that the input targeted (Figure 3B).

Next, we simulated a condition with 10x increased excitation in one dendrite and 10x increased inhibition in the other, resulting in a cell ready to fire in tonic-mode and burst-mode simultaneously in different dendrites. With this setup, we were able to achieve tonic and burst firing by varying the target dendrite of the driver input (Figure 3C). This effect was abolished when the excitation and inhibition were increased uniformly in both dendrites by 10x (Figure 3D), and when only excitation (Figure 3E) or only inhibition (Figure 3F) were increased by 10× in one of the dendrites.

An inspection of the ionic currents activated when the cell received differential inputs reveals that localized de-inactivation of T-channels in distal regions of the dendrites is the main intrinsic mechanism enabling this simultaneous tonic-and burst-ready mode in the cell (data not shown). When one dendrite receives increased excitation, there is local depolarization in that branch, inactivating its local T-channels. Conversely, the branch that receives strong inhibition will experience local hyperpolarization and de-inactivation of its T-channels. Therefore, driver inputs to the depolarized dendrite will trigger stronger Na^+^-K^+^ currents that propagate to the soma leading to tonic firing. Activation of driver inputs on the hyperpolarized branch will result in stronger Ca^2+^ currents, leading to burst firing. Also, continued stimulation will result in continued spiking during tonic firing, but not during burst firing.

In conclusion, our results using this single cell, three-compartment biophysical model suggest that a differential regulation of the membrane potential in thalamic dendrites (depolarization of one dendrite and hyperpolarization of another) could lead to dendrite-specific triggering of bursts in TC neurons. This demonstrates a mechanism by which individual TC cell dendrites selectively modulated by corticothalamic feedback could initiate bursting in response to feedforward inputs.

**Figure 4:**
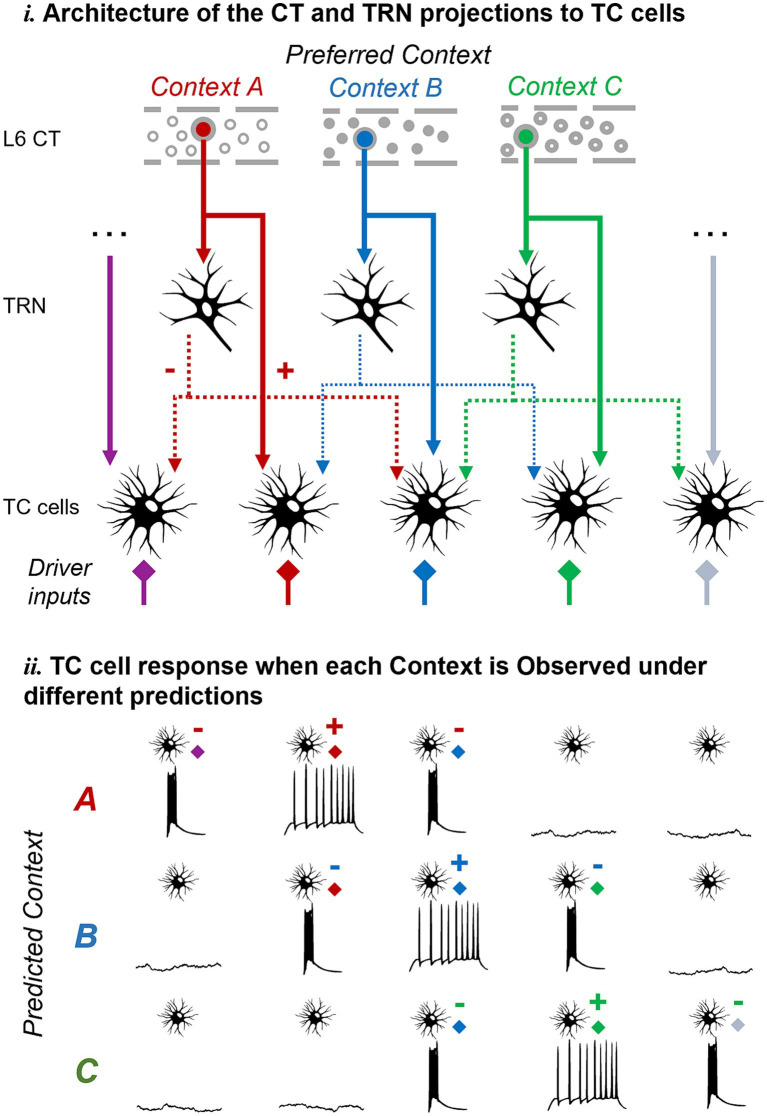
Pictorial representation of the proposed hypothesis and its predictions at the cell population level. The same mechanism proposed at the single-cell level could work at the cell population level to enable TC neurons to discriminate unexpected inputs or contexts as defined by L6 feedback. Abbreviations as in Figure 1.

In addition to input discrimination at the single cell level, L6 CT projections could set different ensembles of TC cells in burst-ready and tonic modes enabling input discrimination at the cell population level. The divergence and convergence of connections within the thalamic circuit can enhance the combinatorial capabilities of such a population-level mechanism, enabling a differential response to the same input feature in the presence of different top-down contexts. A conceptual example is illustrated in Figure 4, showing how L6 CT cells representing different contexts could hyperpolarize various subsets of TC cells. Consequently, this would lead to distinct patterns of burst and tonic firing in the TC population depending on the actual context encountered by the subject.

## 4 Discussion

We have discussed evidence indicating that the functional architecture of the L6 corticothalamic circuit promotes inhibition in TC cells that are functionally misaligned with respect to the L6 cells responsible for such inhibition. We propose that this evidence supports the hypothesis that thalamic bursts provide a mechanism to signal deviance from cortical predictions, a mechanism that could be implemented at the single cell or cell population level. At the single cell level, the hypothesis predicts that differential CT regulation of TC dendrites (inhibition of some dendrites and depolarization of others) would lead to burst or tonic firing in TC cells depending on which driver input, unexpected or expected, becomes active. We demonstrated the biophysical feasibility of this mechanism at the single cell level through modeling, finding that simultaneous excitation and inhibition of different TC dendrites (but not each type of modulation in isolation) can lead to opposite firing modes in response to driver inputs. Combined with the complex patterns of synaptic connectivity resulting from divergent and convergent CT connections within the thalamus, this mechanism could further facilitate top-down, context-specific discrimination of inputs.

### 4.1 Theories of the function of thalamocortical networks

**Figure 5:**
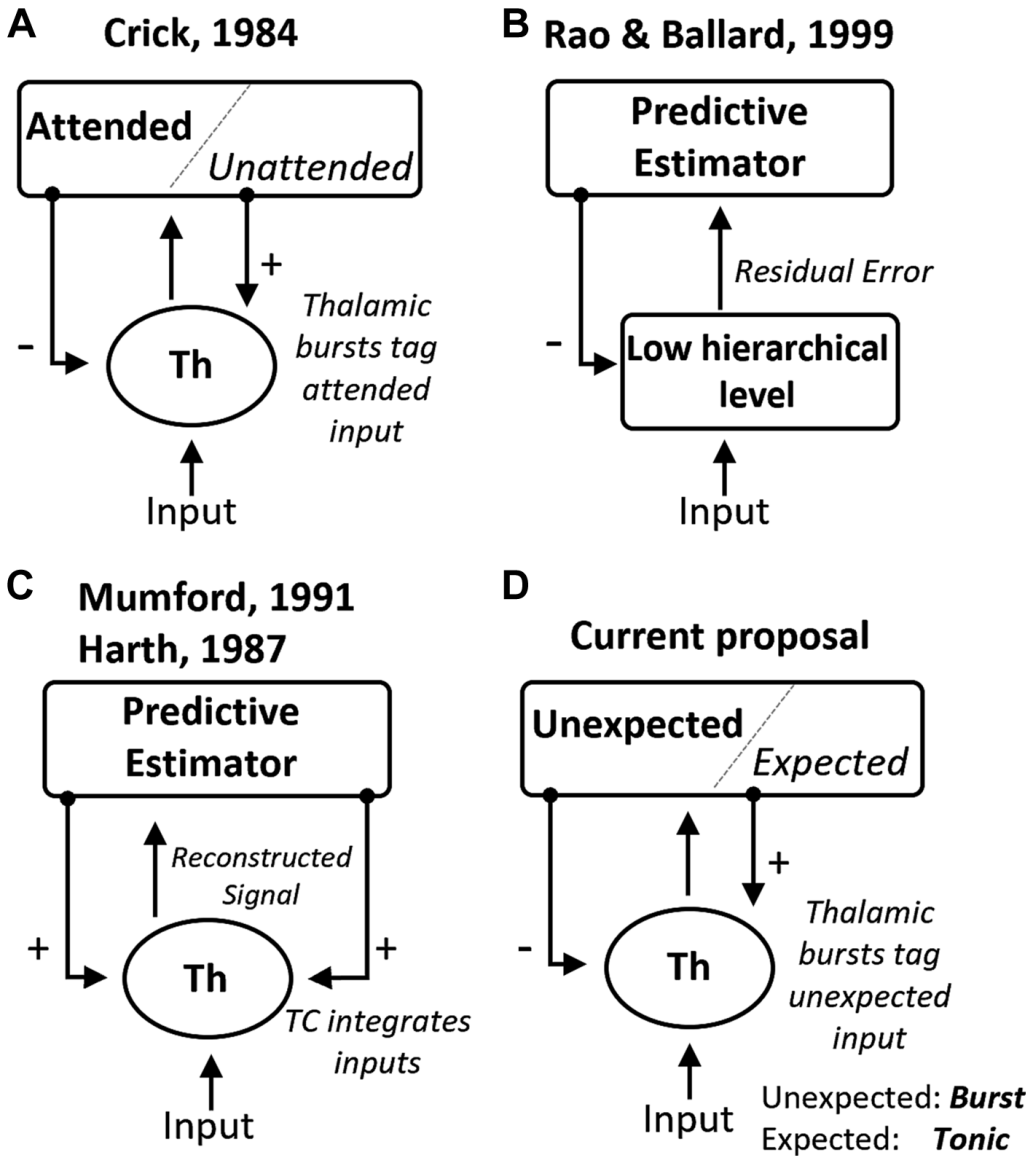
Summary of theoretical mechanisms for the role of the thalamus in predictive processing. **(A)** The “searchlight hypothesis” (Crick, 1984) proposed that bursts in the thalamus, induced by top-down excitation of TRN, could tag attended information transiently. **(B)** General hierarchical network for predictive coding proposed by Rao and Ballard (1999), which suggests that lower-level areas estimate prediction errors after comparing predictions and observations. **(C)** The “active blackboard” hypothesis suggested that the thalamus generates a best guess of the environment through integration (Mumford, 1991) or optimization (Harth et al., 1987) mechanisms. **(D)** Here we review experimental evidence that is consistent with the hypothesis that thalamic bursts indicate input that is unexpected based on cortical predictions.

Our work builds upon theories that proposed a direct role for the thalamus in top-down covert attention and inference (Crick, 1984; Harth et al., 1987; Mumford, 1991). Our study advances these proposals in several ways (Figure 5). The searchlight hypothesis suggested that covert attention manifests as “rapid bursts of firing” in TRN cells (Figure 5A). However, experiments in the visual system (McAlonan et al., 2008) found a relative decrease in TRN firing in attended compared to non-attended locations in the visual field (although increases in TRN activity were observed with attentional shifts across sensory modalities; McAlonan et al., 2006). In these experiments, animals were well-trained in the task and familiar with the presented visual stimuli, allowing for reliable behavior but possibly seeing less unexpected events. What emerges from the results reviewed here is that the activation of TRN may depend on the occurrence of unexpected events, pointing to the need for experiments where animal expectations are systematically manipulated to investigate their correlation with TRN firing modes. Hierarchical predictive processing theories provide a framework to consider top-down expectations in interpreting thalamic cell responses. These theories suggest that early levels of a hierarchical neural system compute prediction errors (Rao and Ballard, 1999; Friston, 2005). That is, that each level in the circuit hierarchy attempts to predict the responses at previous lower levels via feedback connections, while feedforward connections send the unexplained error to the higher level (Figure 5B). However, these theories have been primarily focused on cortical networks, and it is not clear that the same processes operate in the thalamus, given that thalamic inhibition does not necessarily suppress spiking but instead changes the firing mode. This leads us to propose that CT feedback connections from L6 may specifically modulate the TRN to represent unexpected inputs, and as a result, burst firing may occur in TC cells when they receive an input that deviates from cortical expectations. To gain insight into these possibilities, future experiments on attentional modulation in the thalamus should consider the statistical correlations between stimulus features to understand how they relate to thalamic firing modes. For example, if a vertical line is expected with high probability, a horizontal line would be highly unexpected, and this type of orthogonal stimulus properties may be associated with significantly different membrane potentials and firing mode in TC cells.

Another perspective is the one provided by the “active blackboard” hypothesis (Figure 5C), which sees the thalamus as a region that integrates cortical inferences with sensory inputs to generate the “current best reconstruction of some aspect of the world” (Mumford, 1991). This hypothesis shares similarities with predictive processing theories, as cortical feedback represents predictions. However, it assigns a complex, dynamic role to the thalamus, where its output to downstream brain regions represents an integrated reconstruction of the world. Instead, predictive processing frameworks (Figure 5B) may interpret the thalamus as a circuit that generates prediction errors as a result of comparing its inputs against CT predictions. Based on the observations of burst firing in response to novel stimuli (Section 2.2), we propose that this firing mode may play a role in the predictive computations contributed by thalamic circuits. However, rather than signaling error or novelty, bursts may provide a mechanism to estimate deviance from what was anticipated (Figure 5D). To disentangle these possibilities, new experimental designs could compare thalamic burst rates during the presentation of novel stimuli and of familiar stimuli presented within expected patterns and out of context. By comparing these conditions, we can understand the specific contributions of thalamic firing modes and their relationship to novelty, prediction errors, and deviance estimation.

Along these lines, the hierarchical view of brain systems has dominated the interpretation of neuroscience data for decades, being instrumental in advancing our understanding of the function of the forebrain (Felleman and Van Essen, 1991; Yamins and DiCarlo, 2016). Other views of how information is integrated in brain circuits such as Mumford’s blackboard had received less attention (but see Hawkins et al., 2018). Even von Helmholtz’s unconscious inference ideas, which were theoretical and did not imply hierarchical circuit mechanisms (Meyering, 1989), have evolved into that form under modern predictive processing theories (Keller and Mrsic-Flogel, 2018). Hierarchical circuits have garnered strong experimental support, but additional connectivity patterns are becoming apparent. Non-hierarchical circuits may be particularly important in higher order (HO) thalamic nuclei, those connected with higher order sensory and associative cortical regions (Sherman, 2017). HO TC cells project to multiple cortical areas and receive L6 feedback from them (Giguere and Goldman-Rakic, 1988; Killackey and Sherman, 2003; Llano and Sherman, 2008). We do not know if these different L6 inputs converge on individual cells or form parallel processing streams on different TC cells. HO TC and TRN cells also receive multiple drivers from different hierarchical levels, and these drivers do converge in TC cells, which therefore can integrate information across hierarchical levels (Groh et al., 2014; Sampathkumar et al., 2021; Carroll et al., 2022; Casas-Torremocha et al., 2022). Likewise, projections from one sector of the TRN can diverge to multiple nuclei of the dorsal thalamus (Clemente-Perez et al., 2017; Crabtree, 2018; Li et al., 2020), and modulations of subthreshold membrane potential between TRN sensory modalities have been demonstrated (Kimura, 2014). While hierarchical circuits are beneficial in integrating increasingly complex sensory information (e.g., from detailed features to objects in the visual system; Yamins and DiCarlo, 2016), the connections that bridge across thalamocortical hierarchical levels may facilitate inference at more abstract levels (e.g., concepts, rules).

The proposed hypothesis is constrained by the lack of experimental evidence concerning the involvement of L6 and thalamus in predictive processing. It remains unclear if L6 conveys information on expected and unexpected stimuli or contexts. Nevertheless, the open loops formed by branching L6 CT axons, which both excite and inhibit different sets of thalamic cells, are intriguing within the framework of predictive processing. Perhaps the most basic statistical regularity in the environment, or one of the easiest to infer, arises from orthogonal stimulus properties. For instance, stimuli consistently observed as dark (or vertically oriented, or of high temporal frequency) will be highly unexpected when they present the opposite feature, such as being white (or horizontally oriented, or of low temporal frequency). Having evolved under their constraints, sensory systems reflect the statistics of natural environments (Lewicki, 2002; Li et al., 2003; Freeman et al., 2013; Manookin and Rieke, 2023). Therefore, from an evolutionary point of view, it is reasonable to expect that sensory systems have developed a mechanism to exploit this specific type of statistical regularity through a push-pull system for detecting deviance. The same principle applies to higher-order statistics or cognitive levels, although most higher-level statistics may need to be learned in response to dynamic changes in specific environments.

### 4.2 Functional architecture at the single cell level

We used a computational model of a single TC cell with two dendrites to assess basic predictions of the hypothesis in a simplified neuronal architecture. To understand the computations in more complex and realistic circuits, there is a need for experimental data on the convergence and divergence patterns in the thalamocortical circuit at the single cell level. We do not have precise estimates of how many L6 cells converge on individual thalamic cells, or how the response properties of the presynaptic and postsynaptic cells compare to each other.

Topography is a key organizing factor of neuronal response properties both in cortex and thalamus. Anatomical results show that there is a general correspondence between the topographical organization of sensory representations in the cortex and in the thalamus (Deschênes et al., 1998; Rockland, 2021; Shepherd and Yamawaki, 2021). However, it is not completely clear whether the pathways looping between the thalamus and cortex are closed (reciprocal) or open (non-reciprocal) at the single cell level, and with respect to the response properties of the pre-and post-synaptic cells (alignment, misalignment). There is evidence of reciprocal circuits for the direct CT projections to TC cells in several sensory systems (Shepherd and Yamawaki, 2021), but CT axon terminals often spread beyond the thalamic area projecting to L6, suggesting that non-reciprocal loops are present (Rockland, 2019), and the organization at the single-cell level is even less clear regarding the indirect connections via TRN. Also, within a specific cortical location, cell response properties can vary substantially (Sato et al., 2007; Kanold et al., 2014; Rockland, 2019; Wang et al., 2022), which opens the question of how the circuit connections are established with respect to the response properties of pre-and postsynaptic cells, as discussed here. Anatomical and functional data at the single cell level will be key to determine how the alignment and misalignment of functional response properties influence the finer wiring logic and reciprocity of thalamocortical connections.

We do know from electron microscopy studies that TC cells receive about a third of their synapses from L6 inputs (Erişir et al., 1997; Van Horn et al., 2000), and one could imagine a combinatorial code where distinct L6 inputs converge on a thalamic cell (TC or TRN) to regulate its membrane potential depending on the expected likelihood of the combinations of sensory (or more complex) features that the L6 inputs represent. Estimates of L6 convergence onto TC cells suggest that at least 10 L6 cells converge on a TC neuron, up to 100 (Sherman and Koch, 1986). Assuming the minimum of 10 L6 cells, if half of them need to be active to depolarize a TC neuron near the spiking threshold, there are 252 different combinations in which 5 out of 10 cells could be active and represent L6 expectations conveyed to the TC cell in the form of subthreshold depolarization. This mechanism would resemble pattern detection mechanisms proposed in cortical dendrites in which the activation of clusters of NMDA receptors leads to dendritic spikes (Major et al., 2013; Hawkins and Ahmad, 2016). A similar combinatorial code would work on TRN cells, except that the result would be TC hyperpolarization that, according to our proposal, would reflect unexpected feature combinations. In the case of the indirect L6 pathway through TRN, the number of distinct combinations that could be represented as unexpected through subthreshold hyperpolarization would be given by the number of convergent axons from L6 cells onto TRN and from TRN on TC cells, together with the number of active TRN inputs needed to de-inactivate enough T current to produce a burst in a TC neuron. While we lack direct measurements of L6 to TRN convergence, estimates of TRN to TC projections in the visual sector of the TRN (the perigeniculate nucleus, PGN) suggest that the ratio is between10-20 PGN to 1 TC cell, and that each PGN cell produces relatively small IPSPs, less than 2 mV when in tonic mode. Assuming as before that half of the input cells (TRN) need to be active to inhibit the TC sufficiently to de-inactivate the T current, the estimate of potential combinations represented at TRN to TC inputs is similar to those in the L6 to TC projections, except that the indirect pathway would add additional combinations at the L6 to TRN step. Detailed anatomical studies and computational models will be important in figuring out the functional connectivity patterns (convergence, divergence) in the L6-TC circuit and the resulting effect on the membrane potential of individual TC cells.

Another aspect is the spatial distribution of the input synapses on the dendritic tree of TC cells. Data from cortical pyramidal cells demonstrate synaptic architectures that are functionally organized along the dendrites (Sheffield and Dombeck, 2015; Scholl et al., 2017; Kerlin et al., 2019). However, there is limited information regarding the synaptic arrangements on TC dendrites, although there is evidence of dendrite-specific clustering of inhibitory synapses (Crandall and Cox, 2012). Overall, the functional properties of synapses that converge on a given TC dendrite from the L6 direct and indirect inhibitory pathways, and how they relate to the properties of driver synapses are unclear. In the current formulation of the proposed mechanism (Figure 2), we have examined the possibility of dendrite-specific organization with inhibitory synapses that converge on the same dendrite as driver input with similar functional properties (leading to a burst when that driver input is unexpected). However, the mechanism could also be implemented at the cell population level (Figure 4), where individual TC cells (rather than dendrites) may be set in a burst-ready mode based on cortical expectations. In brain slices, the level of depolarization required to evoke a burst by activating individual TC dendrites was substantially greater than that required at the soma (Figure 6 of Connelly et al., 2015). This suggests that burst generation requires multi-dendrite activation of T-channels *in vitro*, but whether dendrite-specific bursts can be triggered *in vivo* remains unknown. Ultimately, characterizing the functional and dendritic organization of synapses from different thalamic inputs will be crucial for understanding how individual or ensembles of TC cells integrate inputs, and whether firing mode non-linearities contribute to computing the output that the thalamus relays to downstream brain regions.

### 4.3 Burst firing in first and higher order TC cells

The general connectivity of thalamocortical circuits is similar across FO (connected to primary sensory areas) and HO (associative and cognitive) thalamus, with the exception that HO TC cells receive a larger proportion of L6 relative to driver synapses, possibly reflecting the integration of information from more cortical areas in HO cells (Van Horn and Sherman, 2007). Interestingly, there are some differences in burst properties between TRN cells connected to FO and HO TC cells that have implications for our proposal. Intracellular recordings suggest that TRN-FO cells produce bursts more reliably than HO cells. That is, when hyperpolarized to the same level, FO cells are more likely than HO to produce bursts when released from inhibition (Kimura et al., 2012; Clemente-Perez et al., 2017; Li et al., 2020; Martinez-Garcia et al., 2020). This could perhaps reflect the fact that, given that sensed data represents the “ground truth,” a FO circuit can be more “certain” about detecting discrepancies between prediction and observation. Consistent with the TRN findings, HO TC cells in somatosensory and auditory thalamus were less likely to burst when released from inhibition compared to FO TC cells, and the HO bursts had slower latencies (Desai and Varela, 2021). It is possible that HO cells only produce a burst if lower-level models that send them predictive input are very ‘certain’ about their predictions. By being less reliable to burst, HO cells could avoid potential errors in lower-level models. In the language of predictive processing theories, burst intrinsic properties (burst propensity, reliability, and latency) could provide mechanisms to implement “precision,” defined as the confidence associated with predictions (Friston et al., 2014). While the higher reliability to produce bursts in FO cells may seem at odds with the observation of higher proportions of bursts in HO cells recorded *in vivo* (Ramcharan et al., 2005; Varela and Wilson, 2020), it is important to keep in mind that comparisons of burst reliability are performed by releasing intracellularly recorded cells from a similar hyperpolarization level to assess burst probability. Instead, burst rates in behaving animals may reflect that cells have different hyperpolarization levels, for example because of top-down modulation by L6 (i.e., FO cells may be more reliable to burst, but they may not burst much unless presented with unexpected stimuli).

While our proposal does not require that bursts *per se* encode specific information about L6 predictions or about the driver input that may trigger a burst, it is possible that burst properties, such as the number of spikes per burst, the latency with which bursts are produced, or the inter-burst interval could provide information about the deviant stimulus or the degree of deviation from L6 predictions. Bursts have in fact been reported to contribute to encoding stimulus features, such as stimulus contrast, transitions from suppressive to preferred stimuli (Reinagel et al., 1999; Alitto et al., 2005; Sanchez et al., 2023), or the degree to which a stimulus matches the preferred properties of the thalamic cell (Martinez-Conde et al., 2002; Massaux et al., 2004).

## 5 Model description

We implemented a conductance-based multicompartmental (one soma and two dendritic compartments) neuron model with the most relevant thalamic voltage-dependent conductances (Equation 1 below) required to capture the essential features of burst and tonic firing of TC cells (Pinsky and Rinzel, 1994; Booth and Rinzel, 1995; Pospischil et al., 2008). The membrane voltage in each compartment was described by:


$$C_{m}\frac{dV}{dt}=-I_{Na}-I_{K}-I_{A}-I_{Leak}-I_{KLeak}-I_{T}-I_{H}$$

Where *V* is the membrane potential, Cm = 1 μF/cm^2^, *I_Na_* and *I_K_* are the sodium and potassium currents responsible for the action potential, *I_A_* the transient potassium outward current, *I_Leak_* and *I_KLeak_* represent passive leak currents, *I_T_* is the low-threshold calcium current responsible for burst firing, and *I_H_* is the hyperpolarization-activated cation current. Table 1 summarizes the compartment geometry and conductance values for each of the ionic currents in the soma and dendrites of the model.

In these models, the voltage-dependent currents are variants of the same generic Hodgkin-Huxley equation (Hodgkin and Huxley, 1952):


$$I_{j}=g_{j}\;m^{M}\;h^{N}\;\left(V-E_{j}\right)$$

where *I_j_* is the product of the maximum conductance, *g_j_*, activation, m, and inactivation, *h*, variables, and the difference between the membrane and reversal potentials (*V* – *E_j_*).

The model was implemented, simulated and analyzed using the NetPyNE multiscale modeling tool (Dura-Bernal et al., 2019) and the NEURON simulation engine (Awile et al., 2022).

## Data availability statement

The original contributions presented in the study are included in the article/supplementary material, further inquiries can be directed to the corresponding author.

## Author contributions

CV: Conceptualization, Funding acquisition, Supervision, Visualization, Writing – original draft, Writing – review & editing. JM: Data curation, Formal analysis, Methodology, Software, Validation, Visualization, Writing – original draft, Writing – review & editing. BK: Formal analysis, Methodology, Writing – original draft, Writing – review & editing. SD-B: Data curation, Formal analysis, Methodology, Supervision, Writing – original draft, Writing – review & editing. SA: Conceptualization, Formal analysis, Visualization, Writing – original draft, Writing – review & editing.
